# Synthesis of Sphingolipids Impacts Survival of *Porphyromonas gingivalis* and the Presentation of Surface Polysaccharides

**DOI:** 10.3389/fmicb.2016.01919

**Published:** 2016-11-29

**Authors:** Zachary D. Moye, Kornelija Valiuskyte, Floyd E. Dewhirst, Frank C. Nichols, Mary E. Davey

**Affiliations:** ^1^Department of Oral Biology, College of Dentistry, University of Florida, GainesvilleFL, USA; ^2^Department of Microbiology, Forsyth Institute, CambridgeMA, USA; ^3^Department of Oral Medicine, Infection and Immunity, Harvard School of Dental Medicine, BostonMA, USA; ^4^Division of Periodontology, Department of Oral Health and Diagnostic Sciences, School of Dental Medicine, University of Connecticut, FarmingtonCT, USA

**Keywords:** sphingolipid biosynthesis, dihydroceramides, capsular polysaccharides, stress response, persistence

## Abstract

Bacteria alter the biophysical properties of their membrane lipids in response to environmental cues, such as shifts in pH or temperature. In essence, lipid composition determines membrane structure, which in turn influences many basic functions, such as transport, secretion, and signaling. Like other members of the phylum Bacteroidetes, the oral anaerobe *Porphyromonas gingivalis* possesses the ability to synthesize a variety of novel membrane lipids, including species of dihydroceramides that are distinct, yet similar in structure to sphingolipids produced by the human host. The role of dihydroceramides in the physiology and pathogenic potential of the human microbiota is only beginning to be explored; yet there is increasing data indicating that these lipids play a role in human diseases, such as periodontitis and multiple sclerosis. Here, we report on the identification of a gene (PG1780) in the chromosome of *P. gingivalis* strain W83 encoding a putative serine palmitoyltransferase, the enzyme that catalyzes the first step in sphingolipid biosynthesis. While we were able to detect dihydroceramides in whole lipid extracts of *P. gingivalis* cells as well as crude preparations of outer membrane vesicles, sphingolipids were absent in the PG1780 mutant strain. Moreover, we show that the synthesis of sphingolipids plays an essential role in the long-term survival of the organism as well as its resistance to oxidative stress. Further, a PG1780 mutant displayed much lower activity of cell-associated arginine and lysine gingipains, yet slightly higher activity in the corresponding culture supernates, which we hypothesize is due to altered membrane properties and anchoring of these proteases to the cell surface. In addition, we determined that sphingolipid production is critical to the presentation of surface polysaccharides, with the mutant strain displaying less K-antigen capsule and more anionic polysaccharide (APS). Overall, we have discovered that, in addition to their role in pathogenicity, the synthesis of sphingolipids is critical to the cellular homeostasis and persistence of this important dental pathogen.

## Introduction

Cell membranes of eukaryotes and prokaryotes are fluid, yet resilient composites of proteins and lipids (Kaiser et al., 2011). A great variety of lipid structures are found in the membranes of cellular organisms, and the stability of the membranes is maintained due to the amphipathic nature of the lipids that comprise it. In the simplest terms, phospholipids consist of hydrophobic long-chain fatty acids capped with a polar head group. The structured association of the hydrophobic tails permits the formation of a basic lipid bilayer, critical for cellular life. Further, these fatty acyl side-chains modulate membrane fluidity and adjustments in the structure of these lipids alter the biophysical properties of the membrane (Los and Murata, 2004). One major class of amphipathic lipids is glycerol phospholipids, consisting of a glycerol platform to which two hydrophobic fatty acid side-chains and a hydrophilic phosphate group, which can be modified by the addition of various functional groups. These lipids are found in the membranes of both eukaryotic and prokaryotic organisms. In addition to glycerol phospholipids, eukaryotic membranes are populated with lipid species characterized by a sphingoid base (or long chain base), an amino alcohol with a hydrocarbon tail, which is attached by an amide bond to a fatty acid chain (Futerman and Hannun, 2004). This class of lipids, called sphingolipids, was first described in Thudichum (1884), who alluded to the curious structure of these lipids and introducing the term “sphingo-” likely in reference to the Sphinx of ancient mythology. Sphingolipids not only serve as structural components of the lipid bilayer, but can also associate with collections of proteins and other lipids, often sterols, to form energetically favorable partitions in the membrane known as lipid rafts (Lingwood and Simons, 2010; Nicolson, 2014). In addition, multiple species of sphingolipids are now known to function as regulators of signaling pathways within human cells (Hannun and Obeid, 2008). Though originally thought to exclusively populate the membranes of eukaryotic organisms, it is now well established that a variety of bacterial species, including many members of the prokaryotic phylum Bacteroidetes, are capable of synthesizing sphingolipids (Olsen and Jantzen, 2001), yet the importance of these unusual lipids in physiology and pathogenic capacity of the human microbiota is only beginning to be explored.

The human oral cavity is inhabited by multiple bacterial species proficient in sphingolipid biosynthesis including *Porphyromonas gingivalis, Tannerella forsythia*, and *Prevotella intermedia* (Nichols, 1998; Nichols et al., 2004, 2011). Of these sphingolipid-producing organisms, the impact of the sphingolipids generated by *P. gingivalis* on human cells has been the most extensively characterized thus far. *P. gingivalis* is an obligate anaerobe that is strongly implicated in the progression of adult periodontal disease (Socransky and Haffajee, 2005). The oral cavities of healthy adults are persistently colonized by a diverse and relatively stable collection of microbes. In periodontal disease, this homeostasis is disrupted, and there is a shift in the composition of the microbiota to higher levels of Gram-negative anaerobes, resulting in chronic inflammation and bone resorption. *P. gingivalis* has evolved a number of virulence determinants that permit it to persist within the oral cavity and elicit disease, including a carbohydrate-rich capsule, novel lipopolysaccharides, a host of proteolytic enzymes (Cugini et al., 2013), and the synthesis of sphingolipids appears to be an additional contributor to the virulence of this organism.

Though similar in structure to those produced by mammalian species, the sphingolipids synthesized by *P. gingivalis* that have been described thus far contain some notable differences that permit them to be distinguished from those generated by the human host (Nichols et al., 2004). For example, one common sphingoid base found in the sphingolipids of mammalian species is sphingosine, which contains a double bond between C4 and C5, and when attached to a fatty acid chain via an amide linkage, this lipid is termed a ceramide (Futerman and Hannun, 2004). However, the sphingoid base of *P. gingivalis*, which can be 17, 18, or 19 carbons in length, is fully saturated and *iso*branched for the odd numbered carbon chains. This sphingoid base is referred to as sphinganine (dihydrosphingosine) or dihydroceramide (DHC) once attached by an amide bond to a fatty acid chain, which in the case of *P. gingivalis* is predominantly 3-hydroxy *iso*branched C_17_ (Nichols et al., 2004). Notably, the distinct DHC platform produced by *P. gingivalis* has also been observed in several additional members of the human microbiome (Nichols et al., 2011). Both eukaryotic and prokaryotic organisms also attach a variety of head groups to their sphingolipids via the hydroxy group attached to the first carbon of the sphingoid base. To date, three species of DHCs have been observed in membrane extracts of *P. gingivalis*, including the unmodified DHC platform, phosphoethanolamine DHC (PE DHC) and phosphoglycerol DHC (PG DHC) (Nichols et al., 2004). PG DHC can also be modified by the addition of another saturated fatty acid, usually *iso*branched C_15_, via an ester linkage to the 3-hydroxy of the fatty acid chain (Nichols et al., 2004). Thus, clear distinctions are apparent between the sphingolipids produced by human and prokaryotic species, owing perhaps to different evolutionary contexts and genome size constraints; however, the basic structure of the bacterial versions of these unique lipids is similar to those found in eukaryotic species suggesting that they may perform similar functions to those associated with their counterparts in eukaryotic systems.

Recent studies have demonstrated that multiple species of bacterially derived DHCs can be detected at numerous sites throughout the human body, including gingival tissue from healthy sites as well as sites with severe gingivitis (Nichols et al., 2011). Therefore, even though the majority of sphingolipid proficient bacteria are confined to the oral cavity and gastrointestinal tract, it appears that DHCs, from lysed cells, invading bacteria or outer membrane vesicles (OMVs) derived from bacteria, are capable of penetrating human tissues and influencing disease progression. For example, *in vitro* studies with purified *P. gingivalis* DHCs, which were shown to be identical to the lipids detected in diseased tissues, determined that these lipids can modulate prostaglandin production, stimulate inflammatory responses in fibroblasts (Nichols et al., 2004), inhibit osteoblast differentiation and function (Wang et al., 2010), promote apoptosis in chondrocytes (Röhner et al., 2012) and endothelial cells (Zahlten et al., 2007), and increase IL-6 secretion in dendritic cells (Nichols et al., 2009). In addition, isolated sphingolipids from *P. gingivalis* have been shown to increase inflammation in a mouse model of autoimmune disease (Nichols et al., 2009).

While much is known about the pathways of sphingolipid synthesis and the impact of these lipids on the physiology of eukaryotic organisms, there have been very few investigations pertaining to the genetics or biosynthesis of sphingolipids by prokaryotes. Perhaps the best-characterized bacterial species capable of producing sphingolipids is *Bacteroides fragilis*, a common commensal found in the gastrointestinal tract of healthy humans. The first step in the synthesis of sphingolipids is the condensation of serine and a long chain fatty acid to yield 3-ketodihydrosphingosine, and this reaction is catalyzed by the enzyme serine palmitoyltransferase (SPT). In *B. fragilis*, it has been predicted that BF2461 encodes a functional SPT (An et al., 2011; Wieland Brown et al., 2013), and the SPT activity of this enzyme has recently been confirmed biochemically (D. J. Campopiano personal communication). When this gene was deleted and the lipid profile of the mutant and parental strain were compared, it was determined that *B. fragilis* produces both unmodified DHC and PE DHC as well as an α-galactosylceramide, which appears to modify immune cell response (Wieland Brown et al., 2013). These and potentially other sphingolipids produced by *B. fragilis* play critical roles in the physiology of the organism. For example, cultures of *B. fragilis* treated with myriocin, in order to inhibit SPT, displayed several alterations to its physiology including reduced survival after temperature and oxidative stress, and cultures treated with this inhibitor were less able to survive long-term incubation (An et al., 2011). Importantly microdomains, enriched with sphingolipids, were absent from the membranes of cells when SPT was inhibited, and these findings led the authors to suggest that lower levels of DHCs in the membrane may destabilize lipid rafts (populated and stabilized by sphingolipids) and hinder the transmission of signals of environmental stress across the membrane (An et al., 2011). Therefore, the production of sphingolipids is critical to the survival of *B. fragilis* and furthermore synthesis likely impacts its interaction with the host due to changes in membrane composition. We have identified a gene (PG1780) in the oral anaerobe *P. gingivalis* strain W83 with high sequence similarity (89%) to the SPT of *B. fragilis*. In this report, we characterize a mutant strain bearing a deletion of PG1780 and subsequently synthesis of all lipid species containing a sphingoid base. Our results demonstrate the importance of sphingolipids in the ability of *P. gingivalis* to persist and maintain membrane homeostasis, which consequently impact its capacity to antagonize the human host.

## Materials and Methods

### Bacterial Strains and Mutant Construction

*Porphyromonas gingivalis* strain W83 and derivatives, including the K-antigen capsule null mutant ΔPG0106, which was generated previously (Davey and Duncan, 2006) were routinely grown on agar plates containing Trypticase Soy Broth (Becton, Dickinson and Company, Franklin Lakes, NJ, USA) supplemented with 5 μg ml^-1^ hemin, 1 μg ml^-1^ menadione, and 5% defibrinated sheep blood (Northeast Laboratory Services, Winslow, ME, USA) and incubated at 37°C in an anaerobic chamber (Coy Lab Products, Grass Lake, MI, USA) with an atmosphere containing 5% hydrogen, 10% carbon dioxide, and 85% nitrogen. When desired, planktonic cultures of *P. gingivalis* were grown in Tryptic Soy Broth (TSB) medium (without dextrose) (Becton, Dickinson and Company, Franklin Lakes, NJ, USA) supplemented with 5 μg ml^-1^ hemin and 1 μg ml^-1^ menadione (TSBHK). A deletion of PG1780 was generated using the NEBuilder HiFi DNA assembly cloning kit (New England BioLabs, Ipswich, MA, USA) as described using the instructions provided by the manufacturer. Briefly, primers (Supplementary Table S1) were designed to generate upstream and downstream products of approximately 1 kb flanking PG1780 as well as an erythromycin resistance gene (*ermF*) obtained from plasmid pVA2198 (Fletcher et al., 1995). Primers were designed to incorporate sufficient overlapping regions at the ends of the products to permit the assembly of the erythromycin gene with the flanking regions of PG1780. Products were generated using Phusion High-Fidelity PCR Master Mix with HF Buffer (New England BioLabs, Ipswich, MA, USA), and equal portions of these products were mixed and incubated with the NEBuilder HiFi DNA assembly cloning kit according to the manufacturer’s instructions. The product of the assembly cloning kit was amplified by PCR (using the Phusion High-Fidelity PCR Master Mix) and used to transform *P. gingivalis* W83 by electroporation. Complementation of ΔPG1780 was achieved by inserting a functional copy of PG1780 into the plasmid pTCOW (Gardner et al., 1996) to produce pTCOW-1780. Plasmid pTCOW-1780 was then transformed into W83 ΔPG1780 by electroporation. The presence of pTCOW-1780 in ΔPG1780 was maintained by supplementing media with 1 μg ml^-1^ tetracycline.

### Bacterial Growth

To monitor growth, cultures of *P. gingivalis* strain W83 and derivatives were inoculated into TSBHK, grown for 24 h and then diluted into fresh TSBHK media. After approximately 12 h of growth (OD_600_ is typically 0.3–0.4), cultures were normalized with pre-reduced TSBHK media to an equal optical density (usually an OD_600_ of 0.35). Throughout exponential phase, the optical density (600 nm) of cultures was routinely recorded and then every 24 h once the cultures entered stationary phase. To monitor the number of viable cells, cultures were serially diluted, and 10 μl of each dilution was spotted on blood agar plates in the anaerobic chamber. After 9 days, plates were removed from the anaerobic chamber and photographed.

### Preparation of Outer Membrane Vesicles

Cultures of *P. gingivalis* were grown in TSBHK for approximately 24 h and used to inoculate 50 ml of TSBHK, and this culture was grown for an additional 24 h. This culture was then added to 450 ml of TSBHK and grown another period of 24 h in order to generate a dense bacterial culture. At this point, the culture was removed from the chamber and centrifuged to collect cells; aliquots of the pellet were obtained and frozen at -20°C in preparation for lipid analysis. Supernates were filtered through a 0.22 μm pore size filter and subjected to centrifugation at 100,000 × *g* for 50 min. These pellets were pooled, suspended in phosphate buffered saline (PBS), filtered through a 0.2 μm syringe filter, and stored at 4°C until lipid analysis was performed.

### Lipid Extraction, Fractionation, and Characterization

Lipids were extracted from bacterial samples using the method of Bligh and Dyer (Bligh and Dyer, 1959) as modified by Garbus et al. (1963). Bacterial samples were dissolved in chloroform:methanol:water (1.33:2.67,1, v/v/v, 4 ml) as previously described (Nichols et al., 2004). The mixture was vortexed at 15 min intervals for 2 h, and the mixture was supplemented with 0.75 ml of chloroform and 0.75 ml of a buffer comprised of 2 N KCl and 0.5 N K_2_HPO_4_. The mixture was vortexed and centrifuged (2000 × *g*) at 20°C for 4 h. The lower organic phase was removed and dried under nitrogen.

### Mass Spectrometry

Lipid samples were dissolved in the neutral HPLC solvent (hexane:isopropanol:water, 6:8:0.75, v/v/v), the samples were centrifuged at 2500 × *g* for 10 min, and the supernates were removed for HPLC-mass spectrometric analysis. The bacterial lipid samples were injected over a normal phase column (Ascentis^®^Si, 3 cm × 2.1 mm, 5 mm, Supelco Analytical) interfaced with an API Qtrap 4000 instrument (Sciex). Neutral HPLC solvent was delivered under isocratic conditions with a Shimadzu LC-10ADvp pump at a flow rate of 100–120 μl min^-1^. Total ion chromatograms were acquired using negative ion mode and a mass range of 100 to 1800 amu, and MS/MS acquisitions used parameters optimized for specific lipid products. Collision energies for negative ion products were typically between -30 and -55 volts depending on the precursor ion under investigation. Negative ion ESI was carried out at -4,500 V, with a declustering potential of -90 V, focusing potential of -350 V, and entrance potential of -10 V. The ion source temperature was maintained at 300°C. Multiple reaction monitoring (MRM) negative ion transitions for the serine dipeptide lipids of *P. gingivalis*, Lipid 654 and Lipid 430, were *m/z* 653.5/381.4 and *m/z* 430.3/382.3, respectively. Low mass (LM) and high mass (HM) phosphoethanolamine dihydroceramides (PE DHC) were monitored by *m/z* 677.5/240.0 and *m/z* 706.2/140.0 ion transitions, respectively. LM and HM unsubstituted PG DHC (unsub PG DHC) lipids were monitored by *m/z* 709.0/171.4 and *m/z* 737.0/171.4 ion transitions, respectively. LM and HM substituted phosphoglycerol dihydroceramides (sub PG DHC) were monitored by *m/z* 933.0/171.4 and *m/z* 961.5 /171.4 ion transitions, respectively. The MRM peaks were electronically integrated for comparison of lipid class recoveries.

### Electron Microscopy

In order to visualize surface polysaccharides, colonies of *P. gingivalis* W83 and derivatives were scraped from blood agar plates and stained with ruthenium red as previously described (Priyadarshini et al., 2013). Transmission electron microscopy and image analysis was performed by the electron microscopy core at the University of Florida.

### Preparation of Autoclaved Extracts

*Porphyromonas gingivalis* strain W83 and derivatives were inoculated into TSBHK and grown for approximately 24 h. Cultures were diluted into fresh TSBHK, grown to mid-exponential phase and normalized to an OD_600_ of approximately 1.0. Cultures were diluted 1/10, and 10 μl aliquots of each culture were spotted on blood agar plates. After 3, 5, and 7 days, cultures were scraped off the plates and placed into cuvettes containing sterile distilled water. Cultures were normalized to an OD_600_ of 0.5. 1.5 ml of the suspension was pelleted and half the supernatant was removed. The cells were resuspended in the final 0.75 ml and autoclaved at 120°C for 30 min. Once cooled, the extracts were centrifuged, and the supernatants were removed and saved for analysis.

### Enzyme-Linked Immunosorbent Assay (ELISA)

Enzyme-linked immunosorbent assays (ELISAs) for the detection of surface polysaccharides were performed as previously described (Bainbridge et al., 2015). Briefly, autoclaved extracts of *P. gingivalis* W83 and derivatives were diluted in 50 mM carbonate/bicarbonate buffer, pH 9.6 and serially diluted in a 96-well plate; plates containing diluted antigen were incubated at 4°C overnight. After washing plates with PBS containing 0.05% Tween 20 (PBS/Tween), a solution of 5% milk powder in PBS was used to block wells for approximately 1 h at room temperature. After washing with PBS/Tween, wells were incubated for 1 h at 37°C with a serotype specific antiserum previously generated against *P. gingivalis* strain W83 (Alberti-Segui et al., 2010), diluted to a concentration of 1:2000 in PBS containing 0.1% Tween 20 and 0.1% bovine serum albumin (PTB), to detect the capsule. Next, wells were washed with PBS/Tween and then incubated for 1 h at 37°C with a goat anti-rabbit IgM-HRP antibody diluted at 1:5000 in PTB. To detect anionic polysaccharide (APS), the monoclonal antibody 1B5 (kindly provided by Michael Curtis, Queen Mary University of London, London, England) was used at a concentration of 1:100, and an anti-mouse IgG-HPR antibody at 1:5000 was used as a secondary. After a final wash with PBS/Tween, wells were incubated with 3,3′,5,5′-Tetramethylbenzidine (Sigma–Aldrich, St. Louis, MO, USA) until sufficient color appeared. The reaction was stopped with an equal portion of 1 M HCl, and the absorbance was recorded at OD_450_.

### Gingipain Assay

The activity of arginine and lysine gingipains was assessed as previously described (Veillard et al., 2012). Briefly, cultures were inoculated into TSBHK, grown for 24 h and diluted into fresh TSBHK. Once the cultures reached exponential phase, they were normalized to OD_600_ = 1.0, and 1 ml of each culture was removed from the anaerobic chamber and centrifuged. The supernates of these cultures were saved, while the pellets were resuspended in assay buffer (200 mM Tris, 5 mM CaCl_2_, 150 mM NaCl, and 10 mM L-cysteine at pH 7.6). Cultures and supernatants were diluted 1/10 in assay buffer and then serially diluted in assay buffer across a 96-well microtiter plate. The initial optical density at 405 nm was recorded, and the plates were placed in a 37°C incubator for 10 min to equilibrate the temperature. *N*-α-benzoyl-L-arginine-*p*-nitroanilide (BAPNA) or *N*-α-acetyl-L-lysine-*p*-nitroanilide (ALPNA) were added to the wells at a final concentration of 1 mM, and the microtiter plates were incubated for 2 h at 37°C. The final optical density (OD_405_) of the wells was recorded, and the difference between the initial and final optical density was reported.

### H_2_O_2_ Assay

Cultures of *P. gingivalis* W83 and derivatives were inoculated in TSBHK, grown for 24 h and diluted into fresh pre-reduced TSBHK. After approximately 12 h of growth, cultures were adjusted to an OD_600_ of ∼0.35 and split into four aliquots. Water or fresh H_2_O_2_ (Fisher Scientific) was placed in the anaerobic chamber and diluted into pre-reduced water. H_2_O_2_ (final concentration of 150, 200, or 250 μM) or water (as a negative control) was added at equal volumes to the cultures. The initial optical density (OD_600_) was recorded, and cultures were monitored over the next 30 h.

## Results

### Deletion of PG1780, Predicted to be a Serine Palmitoyltransferase, Generated a Sphingolipid Null Mutant

**FIGURE 1:**
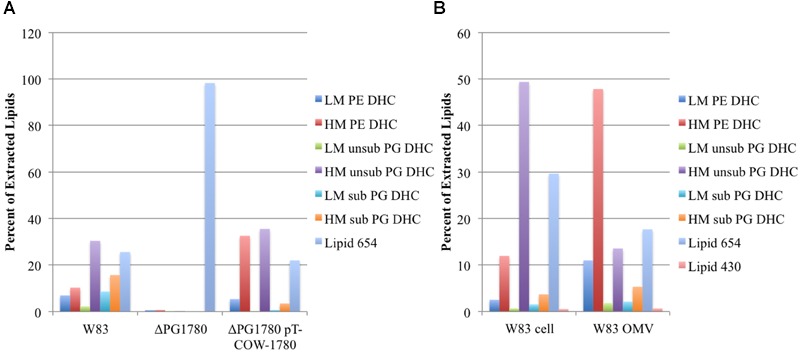
**Deletion of PG1780 renders *Porphyromonas gingivalis* strain W83 unable to synthesis sphingolipids.** Colonies of *P. gingivalis* strain W83, ΔPG1780 and the complemented strain (ΔPG1780 pT-COW-1780) were removed from blood agar plates **(A)**, or *P. gingivalis* strain W83 was grown in TSBHK, cells were pelleted (W83 cell) and the supernate was used to prepare outer membrane vesicles (W83 OMV) **(B)**. Bacterial lipids were extracted from these samples as described in section “Materials and Methods.” Data are representative of three **(A)** or two **(B)** replications showing similar results. The dihydroceramides (DHC) of *P. gingivalis* can be modified by the addition of phosphoethanol (PE) or phosphoglycerol (PG). Further, the sphingoid base ranges in length from 17 to 19 carbons yielding low, intermediate or high mass DHCs. We report here the low (LM) and high (HM) species. In the case of PG DHC, a C_15_ fatty acid can sometimes be found attached to the long chain base, indicated as PG DHCs with (sub) or without (unsub). Finally, lipid 654 and lipid 430 are lipid species that appear in extractions of DHCs but are synthesized by a separate pathway and are not sphingolipids.

Data from previous investigations have suggested an important role for bacterially derived sphingolipids in periodontal disease progression (Nichols, 1998; Nichols et al., 2004, 2012; Nichols and Rojanasomsith, 2006; Wang et al., 2010) and inflammatory autoimmune diseases including a murine model of multiple sclerosis [experimental allergic encephalomyelitis (EAE)] (Nichols et al., 2009). However, the anabolic enzymes critical to the assembly of sphingolipids and their deployment to the membrane in *P. gingivalis* have not been described. SPT is a pyridoxal-dependent enzyme that catalyzes the first step in the biosynthesis of sphingolipids, i.e., the condensation of serine and palmitoyl CoA to generate 3-ketodihydrosphingosine. Initial bioinformatic searches using homologies of this enzyme from mammal sources and then eventually the enzyme encoded by *B. fragilis* (BF2461) (An et al., 2011; Wieland Brown et al., 2013) suggested that *P. gingivalis* contains a gene encoding this transferase (PG1780 in strain W83), which has often been annotated as either 8-amino-7-oxononanoate synthetase or 2-amino-3-ketobutyrate CoA ligase. Our analysis of PG1780 revealed that the enzyme is part of the Conserved Protein Domain Family cd06454 sequence cluster, and this led us to suspect that PG1780 encodes an SPT in *P. gingivalis* strain W83. To test this hypothesis, we replaced PG1780 with an erythromycin resistance cassette via allelic exchange mutagenesis (described in the Materials and Methods section) and analyzed the lipid composition of the resulting mutant. As expected, the mutant strain (ΔPG1780), scraped from blood agar plates, was unable to synthesize dihydroceramides (DHCs) while the parental strain and complemented mutant strain (ΔPG1780 pTCOW-1780) produced comparable levels of these lipid species (**Figure 1A**). To our knowledge, this is the first mutant generated in *P. gingivalis* that is deficient in DHC synthesis. Importantly, the strain lacking PG1780 still maintained high levels of the serine dipeptide lipid 654, which is produced through a separate biosynthetic pathway (Clark et al., 2013).

In addition to extracting lipid samples from *P. gingivalis* growing on plates, we also grew planktonic cultures and looked for the presence of DHCs. As expected, we found a similar profile of DHCs in pelleted cells of *P. gingivalis* W83 grown in liquid culture. We also isolated crude preparations of OMVs from these *P. gingivalis* cultures and found that they were enriched with DHCs (**Figure 1B**) indicating that these lipids are localized to the outer leaflet of the outer membrane.

### Long-Term Survival Is Diminished in a *P. gingivalis* Strain Unable to Produce Dihydroceramides

**FIGURE 2:**
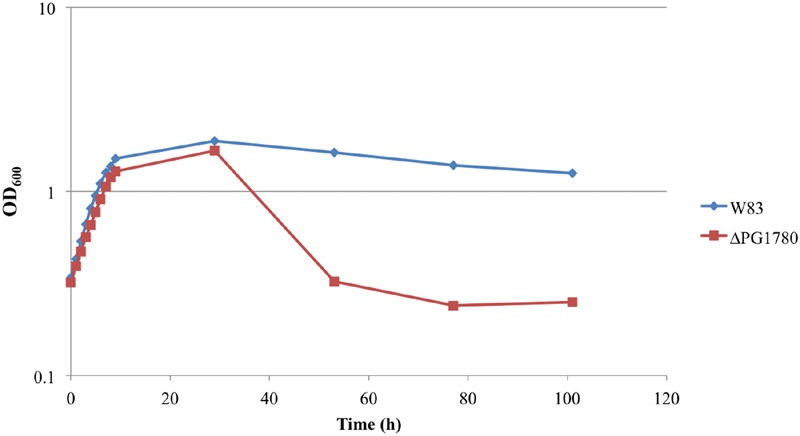
**Growth curve determined that survival of the sphingolipid deficient strain (ΔPG1780) is compromised during stationary phase.** *P. gingivalis* parent strain W83 and the ΔPG1780 mutant (deletion of a serine palmitoyltransferase) were grown in TSBHK media. Data are representative of three replications showing similar results.

In previous studies of *B. fragilis*, it was demonstrated that a reduction in DHC production, due to inhibition of SPT using myriocin, diminished the long-term survival of the organism (An et al., 2011), i.e., the optical density of the treated and untreated cultures remained comparable throughout stationary phase but there was a pronounced decrease in the number of viable cells recovered from the cultures treated with myriocin. Based on these results, we attempted to determine the impact of deleting PG1780 on the growth and long-term survival of *P. gingivalis*. In growth experiments, we found that ΔPG1780 grew at a slightly slower rate to the parental strain (a doubling time of 4.4 h vs. 3.9 h, respectively). Based on the results observed in *B. fragilis*, we also monitored the long-term survival of ΔPG1780 compared to the parental strain. Interestingly, we noted that after approximately 30 h the optical density of the mutant dropped off sharply and continued to diminish over time while the turbidity of parental strain decreased only slightly over the course of the experiment (**Figure 2**). To demonstrate that this decrease in optical density represented the lysis of bacterial cells and not simply auto-aggregation, we serially diluted cultures, spotted 10 μl of dilutions on blood agar plates and incubated the plates for 9 days (Supplementary Figure S1). As expected, the number of colonies recovered from ΔPG1780 decreased dramatically after 30 h while recovery from the parental strain remained relatively stable. We also noted that isolated colonies recovered from ΔPG1780 were much smaller and more pleomorphic than the parental strain. In addition, the parental strain was able to clear much more of the sheep red blood cells (as evidenced by the clear halo around the colonies) than the sphingolipid deficient mutant.

### Deletion of PG1780 Impacts Cell Surface Properties of *P. gingivalis*

**FIGURE 3:**
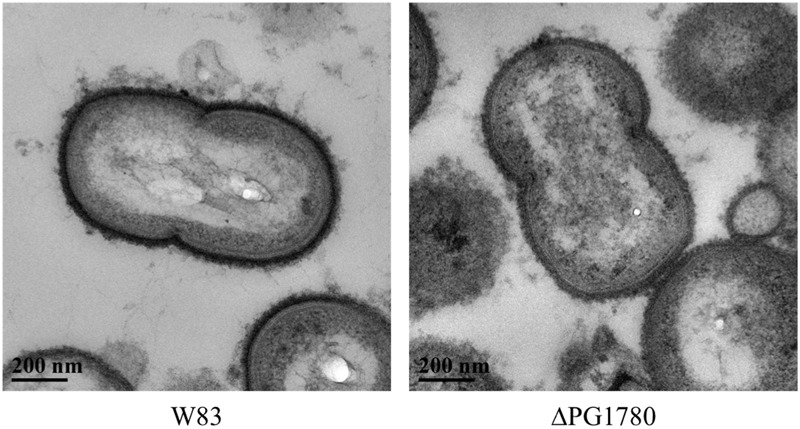
**A sphingolipid deficient strain (ΔPG1780) produces less surface polysaccharide than the parent strain *P. gingivalis* W83.** Cells were stained with ruthenium red as described in the section “Materials and Methods” and viewed by transmission electron microscopy. Once stained, the surface polysaccharides appear as a dense layer surrounding the cell membrane. We consistently observed much lighter staining around the surface of ΔPG1780, indicating that this mutant produces lower levels of polysaccharide at the cell surface.

As indicated, lipid composition influences membrane structure, and thereby function. Since bacterial capsules are typically anchored to the cell surface, we hypothesized that the DHCs could play a role in the presentation of capsule (or another surface polysaccharide) on the cell surface. To explore this hypothesis, we examined ΔPG1780 for changes in the presentation of surface polysaccharides by staining the cells with ruthenium red and imaging with transmission electron microscopy, as previously described (Priyadarshini et al., 2013). As shown in **Figure 3**, the ΔPG1780 mutant lacks the dense layer of polysaccharide at the cell surface, indicating that synthesis of DHCs impacts the presentation of surface polysaccharides.

**FIGURE 4:**
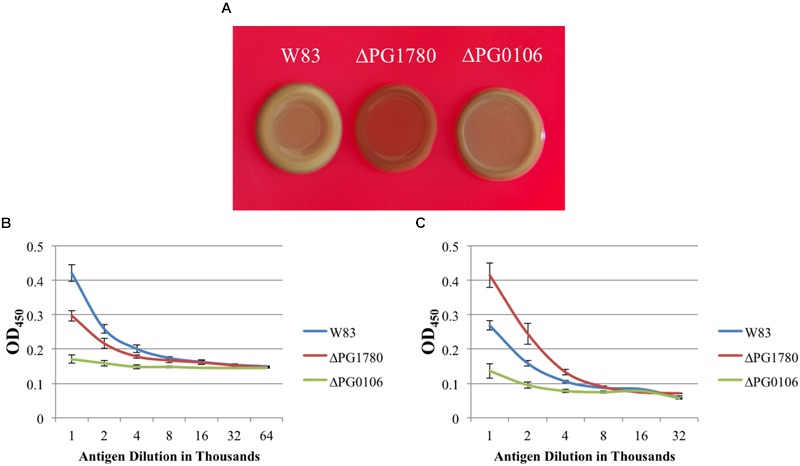
**The surface polysaccharides of *P. gingivalis* are altered in a strain unable to produce sphingolipids.** *P. gingivalis* W83, ΔPG1780 and ΔPG0106 (a strain that lacks the K1 capsule) were grown to mid-exponential phase in TSBHK, normalized, diluted 1/10 and spotted in 10 μl aliquots on blood agar plates. After 5 days, plates were removed from the anaerobic chamber and photographed **(A)**. Colonies were removed from these plates, normalized by optical density and autoclaved to generated antigen. Enzyme-linked immunosorbent assays (ELISAs) were performed to detect K1 capsular polysaccharide **(B)** and anionic polysaccharide (APS) **(C)**. Plates grown to 3 or 7 days were also photographed and used to generate antigen for ELISAs, and both additional time points showed similar results. Data represent the average of three biological replicates with error bars indicating the standard deviation. *P* < 0.05 (by the Student *t*-test) was observed when W83 and ΔPG1780 were compared at antigen dilutions between 1,000–2,000 **(A)** and 1,000–4,000 **(B)**.

Based on these electron micrographs, we began analyzing specific species of surface glycans to determine if any were altered due to the mutation of PG1780. To accomplish this, we grew ΔPG1780, ΔPG0106 (capsule null strain) and the parental strain to exponential phase and then diluted cultures 1/10 and spotted 10 μl of each culture on blood agar plates to simulate the natural state of the bacteria, i.e., in biofilms. After 3, 5, and 7 days, we removed plates from the chamber and observed the colony morphology. The images of bacterial colonies and ELISA results looked similar for samples collected on days 3, 5, and 7; therefore, we show here only the results from day 5. Interestingly, colonies of ΔPG1780 appeared very different from ΔPG0106 and the parental strain (**Figure 4**). Colonies of ΔPG1780 were consistently much darker and thinner than the other two strains, agreeing with our observations during the long-term survival experiments (Supplementary Figure S1). To analyze the carbohydrates presented on the surface of the cell, colonies of all three strains were scraped from these plates, normalized by optical density and autoclaved. ELISAs was performed with these antigens using the αW83 antisera to assess the K-antigen capsule and 1B5 to determine the levels of APS. As expected, we found that ΔPG0106, which was used here as a negative control, gave very little cross reactivity to the capsule antisera and low levels of APS (**Figure 4**). Interestingly, we found that ΔPG1780 produced less capsule polysaccharide than the parental strain; however, the levels of polysaccharide were nowhere near as low as ΔPG0106 (**Figure 4**). Furthermore, the APS presented by ΔPG1780 was higher than the parental strain and ΔPG0106. Overall, these initial studies indicate that the surface glycans presented by *P. gingivalis* are influenced by the presence of sphingolipids. Further biochemical studies are underway to obtain a more detailed analysis of the impact of sphingolipids on the presentation of surface glycans.

### Levels of Gingipains Are Dramatically Altered in a Strain Lacking Sphingolipids

**FIGURE 5:**
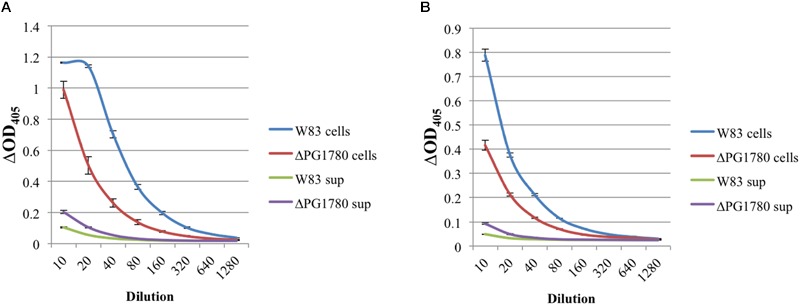
**The proteolytic activity of gingipains is lower in a strain of *P. gingivalis* that is unable to produce sphingolipids.** *P. gingivalis* W83 and ΔPG1780 were grown to mid-exponential phase in TSBHK media, and a gingipain assay was performed on these cultures as described in the section “Materials and Methods.” Cultures were centrifuged, the supernatant was removed and saved while the pellet was resuspended in assay buffer. The bacterial cells (cells) and supernatant (sup) were diluted 1/10 and then serially in assay buffer. After reading the initial optical density (OD_405_I), samples were treated with either *N*-α-benzoyl-L-arginine-*p*-nitroanilide (BAPNA) and *N*-α-acetyl-L-lysine-*p*-nitroanilide (ALPNA) to assess the arginine **(A)** or lysine **(B)** gingipain activity, respectively. Samples were incubated for 2 h, and the final optical density (OD_405_F) was recorded. Results are reported as ΔOD_405_, that is OD_405_F – OD_405_I. Data represent the average of three biological replicates with error bars displaying the standard deviation. *P* < 0.05 (by the Student *t*-test) was observed when W83 cell and ΔPG1780 cell were compared at dilutions between 10–1280 **(A)** and 10–320 **(B)**. *P* < 0.05 (by the Student *t*-test) was observed when W83 sup and ΔPG1780 sup were compared at dilutions between 10–160 **(A)** and 10–80 **(B)**.

The arginine and lysine gingipains are major virulence determinants of *P. gingivalis* and can be anchored to the surface of the cell, released via outer membrane vesicles, or secreted. Due to the lower level of proteolysis observed by the mutant on blood agar plates (data not shown), we next investigated whether the deletion of PG1780 impacted the activity of arginine and lysine gingipains. To monitor gingipain activity, *P. gingivalis* W83 and ΔPG1780 were grown to mid-exponential phase. The cells were pelleted, the supernatant was removed, and the activity of cell-associated and cell free gingipains was determined as described in the section “Materials and Methods.” We determined that the mutant strain produced substantially lower arginine and lysine gingipain activity in the whole cell fraction when compared to the parental strain (**Figure 5**). Further, ΔPG1780 consistently produced slightly more supernate-associated gingipain activity than the parental strain, potentially indicating that the lower level of cell-associated gingipains may be due to an increased release of gingipains into the supernatant, perhaps as a result of changes to membrane composition.

### Deletion of PG1780 Reduced the Ability of *P. gingivalis* to Survive Oxidative Stress

**FIGURE 6:**
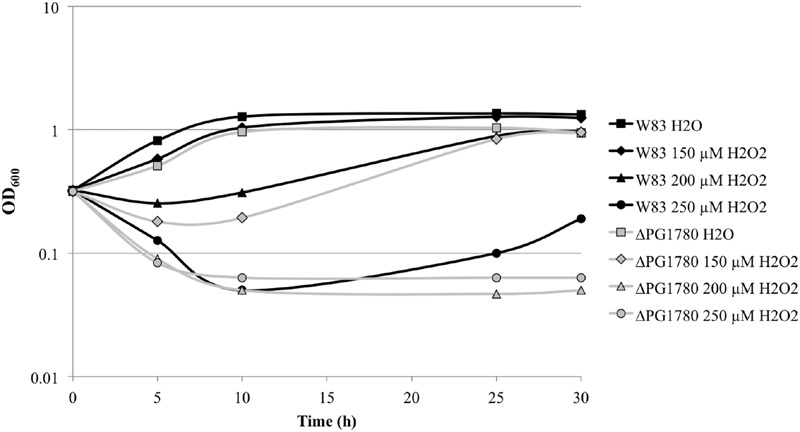
**A sphingolipid deficient strain of *P. gingivalis* is more sensitive to oxidative stress.** *P. gingivalis* W83 and ΔPG1780 were grown to early exponential phase (OD_600_ = 0.35) in TSBHK media. Cultures were normalized, separated into four aliquots and treated with H_2_O (as a negative control) or fresh H_2_O_2_ (at 150, 200, or 250 μM). The optical density of cultures were then monitored for 30 h.

During our characterization of ΔPG1780, we consistently observed that the mutant performed poorly after exposure to oxygen, even for very short intervals (For example, the parental strain and ΔPG1780 were serially diluted and spotted in the anaerobic chamber during our long-term survival assays and grown for 9 days. When strains were diluted and plated outside the chamber, the mutant took substantially longer to grow on blood agar plates). To determine if deletion of PG1780 impacts the ability of *P. gingivalis* to survive oxidative stress, we grew ΔPG1780 and the parental strain to early exponential phase and treated cultures with 150, 200, or 250 μM hydrogen peroxide or water as a control. We found that the parental strain was able to survive the addition of all concentration of hydrogen peroxide we tested. Cultures treated with the highest concentration (250 μM) decreased in density after the initial administration of H_2_O_2_ but were able to recover and resume growth after approximately 30 h (**Figure 6**). In contrast, cultures of ΔPG1780 treated with 200 or 250 μM H_2_O_2_ were quickly killed and were not able to recover after 30 h. Cultures of the mutant treated with the lowest concentration of H_2_O_2_ (150 μM) did initially decrease in density after treatment but were able to recover by the end of the experiment. Thus, overall, ΔPG1780 is much more sensitive than the parental strain to treatment with H_2_O_2_.

## Discussion

Far from acting solely as structural components of mammalian membranes, it is becoming increasingly evident that sphingolipids can serve a wide range of functions within cells, including partitioning into functional microdomains populated by other lipid species and integral membrane proteins (lipid rafts), as well as acting as signaling molecules for a diverse array of cellular processes (Hannun and Obeid, 2008; Nicolson, 2014). In light of the numerous, critical cellular functions associated with sphingolipids, it is not surprising to find that pathogenic microorganisms have evolved mechanisms to manipulate and interfere with sphingolipid signaling in order to promote their pathogenicity (Heung et al., 2006). Moreover, although sphingolipids were once thought to be exclusively produced by eukaryotes, it is now apparent that prokaryotes can also synthesize lipids containing a sphingoid base. In fact, several bacterial species belonging to the human microbiome are capable of producing sphingolipids with many of these species belonging to genera within the phylum Bacteroidetes, representing strict anaerobes that are symbiotic inhabitants of the oral cavity as well as the gastrointestinal tract (Olsen and Jantzen, 2001). Very little is known regarding the function of bacterially derived sphingolipids, yet data indicate that they may function in similar ways to the sphingolipids of eukaryotic cells. For example, the sphingolipids produced by *B. fragilis* have been shown to form microdomains in the membrane, which have been suggested to serve as a means of relaying signals of environmental stress (An et al., 2011). It has also been demonstrated that certain sphingolipids synthesized by *B. fragilis* can serve as signals to subvert the host immune response by modifying the host lipid environment to negatively regulate (dampen down) the immune response, thereby mediating a symbiotic association between the microbiota and the host (An et al., 2014). Hence, a better understanding of why and when these unique lipids are produced may enlighten us as to how microbial communities engage in mutualistic relationships with the host and what changes lead to pathogenicity.

Although bacterially derived sphingolipids are found in both healthy and diseased gingival sites, it was notable that the types of DHCs detected in diseased periodontal tissues was different than for healthy controls (Nichols and Rojanasomsith, 2006; Nichols et al., 2011). These results suggest that disease progression is not associated with simply the presence of sphingolipids, but that shifts occur in the species of DHCs released by the periodontal microbiota in the gingival tissue and blood of patients with periodontitis when compared with healthy controls (Nichols et al., 2011). Another layer of complexity is added by the results of *in vitro* studies using purified fractions of the two major DHCs produced by *P. gingivalis*; these studies revealed that PE and PG DHCs had different effects on eukaryotic cells, due most likely to the polar head group they possess. For example, PG DHCs alter fibroblast morphology and increase prostaglandin E2 production after IL-1β treatment (Nichols et al., 2004) and promote apoptosis in endothelial cells (Zahlten et al., 2007) as well as chondrocytes (Röhner et al., 2012). Both whole lipid extracts of *P. gingivalis* as well as purified PG DHC interfered with bone mineral deposition and osteoblast function, while PE DHC showed little effect (Wang et al., 2010). In parallel, PE DHCs were much more effective at inducing autoimmunity in a murine model of multiple sclerosis [called allergic encephalomyelitis (EAE)] (Nichols et al., 2009). Yet, both DHC classes generally appear to induce inflammatory responses in disease models and likely engage host cell receptors (or groups of receptors) via their polar head groups. Further complicating matters is the fact that DHCs from *P. gingivalis* are presented to the host as a mixture with a number of other DHCs from other bacteria also proficient in synthesis. It is becoming clear that, like the bacteria that produce them, the sphingolipids of *P. gingivalis* are present in both healthy and diseased sites, and shifts in the community of bacteria depositing them or perhaps environmental cues that modulate the synthesis of these unique lipids may contribute to disease progression.

To begin to gain insight into the possible functions of these sphingolipids in regards to *P. gingivalis* physiology, we generated a sphingolipid null mutant; specifically, we deleted a gene predicted to encode a SPT, PG1780 in strain W83, which is the enzyme that catalyzes the first committed step in the sphingolipid biosynthetic pathway. We predicted that the deletion of PG1780 would render *P. gingivalis* completely deficient for the production of sphingolipids and indeed our lipid analysis confirmed this hypothesis (**Figure 1**). Studies on the intestinal strain *B. fragilis*, a close relative of *P. gingivalis*, using myriocin to inhibit SPT, determined that sphingolipid synthesis affects survival of this bacterium during stationary phase, but not the growth of the bacteria during log phase (An et al., 2011). We demonstrated that this is also true for *P. gingivalis*; however, unlike *B. fragilis* treated with myriocin, we were surprised to find that the cells of the *P. gingivalis* sphingolipid mutant lysed, and cultures of the mutant visibly decreased in density during stationary phase (**Figure 1** ; Supplementary Figure S1). The discrepancies in our results may be due to the use of inhibitors in the *B. fragilis* experiments rather than a mutant devoid of sphingolipids. In fact, the authors noted that treatment with myriocin only effectively blocked the synthesis of two-thirds of the DHCs (An et al., 2011). Alternatively, this could indicate a distinction in the function of these lipids in these two different genera. Species of *B. fragilis* possess much larger genomes than *P. gingivalis* (more than two times greater) and may encode additional systems capable of compensating for a loss of DHCs. In either case, cell lysis suggests that there may be conditions during stationary phase, e.g., nutrient stress or build up of a metabolic by product, that lead to the up-regulation of sphingolipid biosynthesis in *P. gingivalis* (and perhaps also *B. fragilis*), and these lipids then play an essential role in stabilizing the cell membrane. Further studies are underway to determine the environmental cues that regulate differential expression of PG1780 as well as other genes involved in the sphingolipids biosynthesis, and additionally, we are working to determine the levels of the different types of lipids under various growth conditions and environmental stresses. Finally, we noted that the growth of ΔPG1780 on blood agar plates was much slower and colonies were much thinner than colonies of the parental strain (Supplementary Figure S1). *P. gingivalis* strain W83 does not form a strong biofilm (Davey and Duncan, 2006), but in contrast to a planktonic culture, growth on the surface of a blood plate may more accurately model the environment of subgingival plaque. Our results suggest that growth of ΔPG1780 is compromised on solid surfaces, and additional studies using more heavily fimbriated strains of *P. gingivalis* (W50, 33277, or 381) could provide a greater understanding regarding the importance of sphingolipids in the colonization and/or invasion of this organism. Importantly, the orthologs of PG1780 in these other strains show 99% identity.

Our initial interest in sphingolipid synthesis began during an investigation of a trans-acting anti-sense RNA (asRNA) positioned within a 77-bp inverted repeat element and located just upstream of an operon involved in production of the K1 capsular polysaccharide in *P. gingivalis* strain W83 (Bainbridge et al., 2015). A bioinformatic search revealed that multiple sites bearing a high degree of sequence identity to the asRNA are located throughout the genome of *P. gingivalis*, and one of these regions was found near PG1786 (data not shown), a gene located just downstream of PG1780. Some of the findings from our current study suggest that sphingolipids may play a role in the deployment and/or anchoring of surface polysaccharides to the outer membrane. The precise identity of the lipid moiety responsible for covalently anchoring capsular polysaccharides to the outer membrane of bacteria has only been definitively identified in a few species. For example, Willis et al. (2013), in a recent report, have observed the direct covalent attachment of capsular polysaccharide to a glycerol phospholipid species containing a lyso-phosphatidylglycerol motif. The proximity of PG1780 to a potential target of a trans-acting anti-sense RNA as well as the altered surface polysaccharides of in ΔPG1780 make it tempting to speculate that sphingolipids could potentially play a role in anchoring surface polysaccharides to the outer leaflet in *P. gingivalis.* Another striking discovery from our study was that ΔPG1780 mutant displayed an overall decrease in the amount of surface polysaccharides, with a shift to low levels of K-antigen and higher levels of APS. Our current working hypothesis is that the synthesis of higher levels of APS may be either an indirect effect, i.e., a compensatory mechanism in response to the lack of sphingolipids; or K-antigen capsule and APS may be inversely regulated. We are currently taking a biochemical approach to determine the carbohydrate composition of the sphingolipid mutant compared to the parent strain to further clarify our results.

*Porphyromonas gingivalis* is known to be highly proteolytic, with major activity from a class of endopeptidases known as gingipains. These proteases are essential to nutrient acquisition and are located on the cell surface and released into the surroundings (Curtis et al., 1999a). APS is immunologically connected to the post-transcriptional modification of Arg-gingipains (RgpA), since a monoclonal antibody (MAb 1B5) raised against RgpA, cross reacts with APS (Curtis et al., 1999b; Paramonov et al., 2005). The current model is that Arg-gingipains are covalently anchored to APS, suggesting that their synthesis would be coordinated; yet our data indicate that the synthesis of APS and gingipain activity are not necessarily linked, since APS was detected at higher levels in the PG1780 mutant, yet gingipain activity was down. To our knowledge this the first report of a *P. gingivalis* mutant strain that over-produces APS.

**FIGURE 7:**
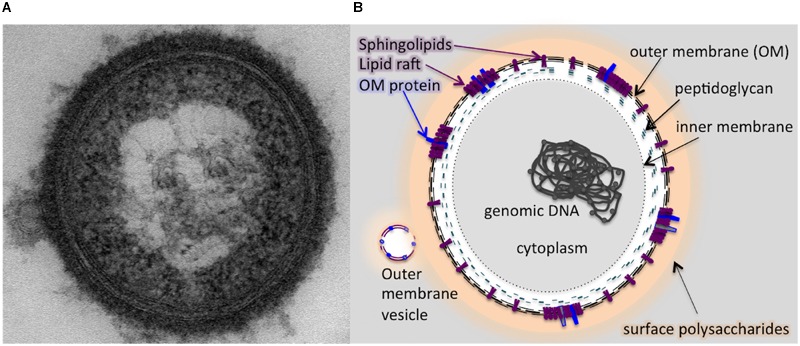
**A schematic model illustrating the potential localization of sphingolipid in the membranes of *P. gingivalis* based on the results of our study.** The outer membrane of *P. gingivalis* contains sphingolipids and the cell is cloaked in capsular polysaccharides. **(A)** Transmission electron micrograph (TEM) of a *P. gingivalis* cell (encapsulated strain W83) stained with ruthenium red, showing a dense layer of surface polysaccharides and release of an outer membrane vesicle (OMV). Graphic representation of the TEM image **(B)** indicating localization of sphingolipids in the outer membrane (OM) along with select OM proteins co-localized with dense areas of sphingolipids, representing lipid rafts. The membrane of the OMV is shown to contain sphingolipids as well as the lipid-associated OM proteins. The OMV is also coated with surface polysaccharides (LPS, APS, and K-antigen capsule).

It is also important to note that we were able to recover DHCs from our outer membrane vesicle preparations of *P. gingivalis* strain W83, which would seem to indicate that DHCs are present in the outer leaflet of this organism. As discussed above, sphingolipids are able to partition into membrane subcompartments with other lipids, often sterols, and integral membrane proteins referred to as lipid rafts. Investigations in *B. fragilis* revealed that inhibition of sphingolipid production resulted in a complete loss of lipid rafts, and it is probable that deletion of PG1780 could result in the destabilization of lipid rafts in *P. gingivalis*. Though speculative, we suggest that lipid rafts in the outer leaflet could facilitate the efficient transport or localization of some species of surface polysaccharides (or potentially gingipains) to the cell surface, and without sphingolipids, the protein components of lipid rafts may be destabilized, leading to inefficient transport. Overall, our results suggest that DHCs are present at the outermost surface of the bacterial cell, and based on our results and previous work with sphingolipids, we suggest a model whereby sphingolipids may serve to regulate surface polysaccharides or attach/deliver them to the outer surface of the cell (**Figure 7**).

## Conclusion

We have uncovered the first enzyme in the pathway for generating sphingolipids in the oral pathobiont *P. gingivalis*. It has been well established that DHCs derived from this bacterium contribute to its virulence, and the results of our study demonstrate that these unique lipids are also critical to the long-term persistence and presentation of other virulence attributes, such as gingipains and capsular polysaccharide. As mixed populations of bacterially derived sphingolipids can be detected at both sites of health and disease, it is important to determine what factors drive the synthesis pathways toward the production of the different types of sphingolipid species, especially those enriched at diseased sites. Further investigations into the regulation of sphingolipid biosynthesis in the Bacteroidetes and the specific enzymes involved in their production will likely increase our understanding of the balance between commensalism and disease.

## Author Contributions

KV contributed to data acquisition, analysis, and interpretation. ZM contributed to the conception, experimental design, data acquisition, interpretation of data sets, drafting and critically reviewing the manuscript. FD predicted that PG1780 encoded serine palmitoyltransferase and contributed to conception and interpretation of data. FN contributed to acquisition, analysis, and interpretation. MD contributed to the conception, experimental design, interpretation of data, drafted and critically edited the manuscript.

## Conflict of Interest Statement

The authors declare that the research was conducted in the absence of any commercial or financial relationships that could be construed as a potential conflict of interest.
