# Supplementary material for: Synthesis of Sphingolipids Impacts Survival of Porphyromonas gingivalis and the Presentation of Surface Polysaccharides
Source: Front Microbiol. 2016 Nov 29;7:1919. doi: 10.3389/fmicb.2016.01919 (PMC5126122; doi:10.3389/fmicb.2016.01919)
Supplement: Supplementary file 1 [file Table_1.DOCX]

**Table S1. List of Primers**

| US1780 F | TATGCCGTTTGGGATGGC | For ΔPG1780 with ErmF |
| --- | --- | --- |
| US1780 R | cttttttgtcatTAGCCTCGAGAGTTAGCTTATTAGG | For ΔPG1780 with ErmF |
| ErmF1780 F | cgaggctaATGACAAAAAAGAAATTGCC | For ΔPG1780 with ErmF |
| ErmF1780 R | attacacttaCTACGAAGGATGAAATTTTTC | For ΔPG1780 with ErmF |
| DS1780 F | cttcgtagTAAGTGTAATTAATGATAACAGCGTGTTG | For ΔPG1780 with ErmF |
| DS1780 R | AGCACAGCCACAGCAGCA | For ΔPG1780 with ErmF |
| groES1780F | gtcaggcaccgtgtaATTGGATAGATGCCCTGC | For making pTCOW-1780 |
| groES1780R | ttttcccatTGTTGCTTGGTTTGTTATTG | For making pTCOW-1780 |
| PG1780_groES/pT-cow F | caagcaacaATGGGAAAATTGTTACAGGATAAATTG | For making pTCOW-1780 |
| PG1780_groES/pT-cow R | gtgccgccggcttccattCAGGACACCGTTCTGCTTG | For making pTCOW-1780 |
