# Supplementary material for: Synthesis of Sphingolipids Impacts Survival of Porphyromonas gingivalis and the Presentation of Surface Polysaccharides
Source: Front Microbiol. 2016 Nov 29;7:1919. doi: 10.3389/fmicb.2016.01919 (PMC5126122; doi:10.3389/fmicb.2016.01919)

**Figure S1.** A sphingolipid deficient strain (∆PG1780) of *P. gingivalis* is less viable in stationary phase. During the growth study discussed in the text (See Figure 1), cultures of *P. gingivalis* W83 and ∆PG1780 at 29, 53, 77 and 101 hours, *i.e*. 24 hours apart, were serially diluted, and 10 µl of each biological replicate was spotted in duplicate on blood agar plates. In all cases, each dilution from 10^-1^ to 10^-8^ was spotted. After 9 days, the plates were removed from the anaerobic chamber and photographed.


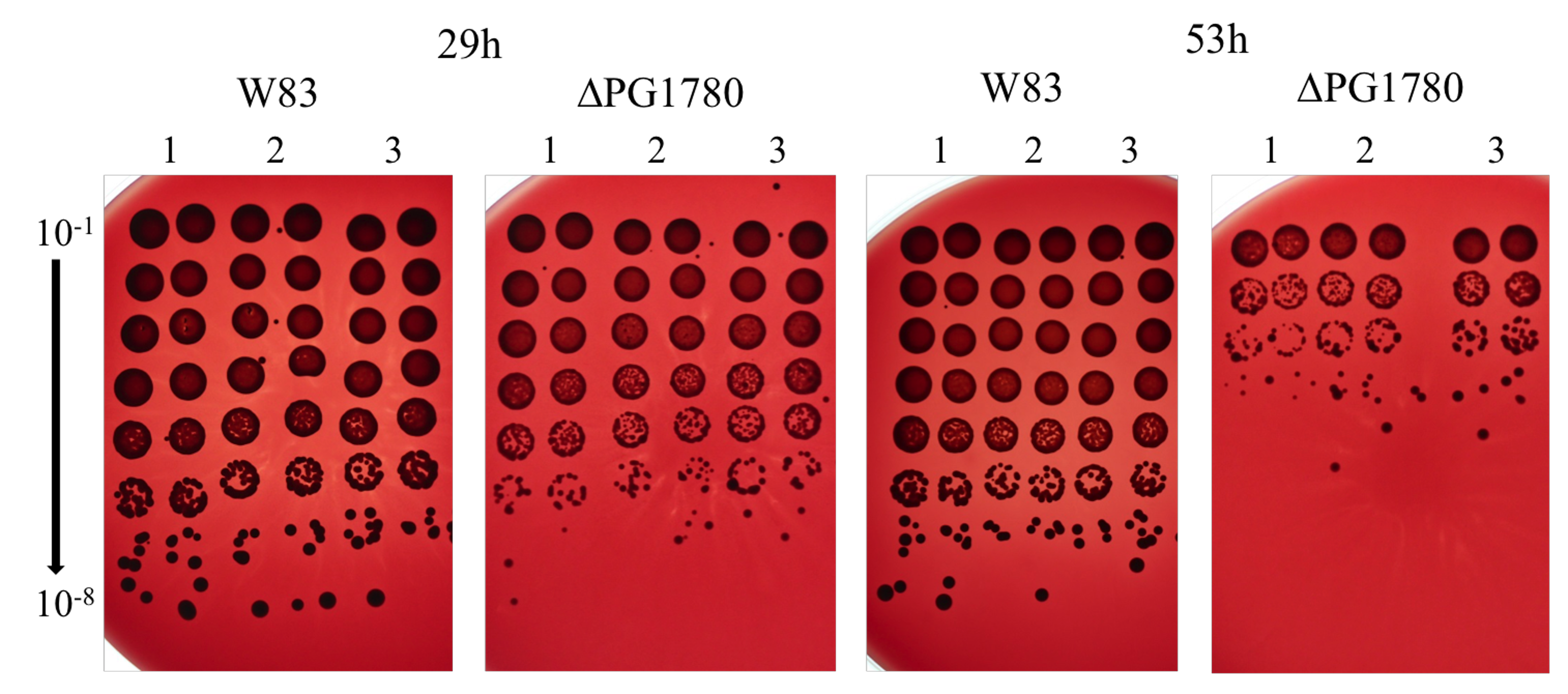


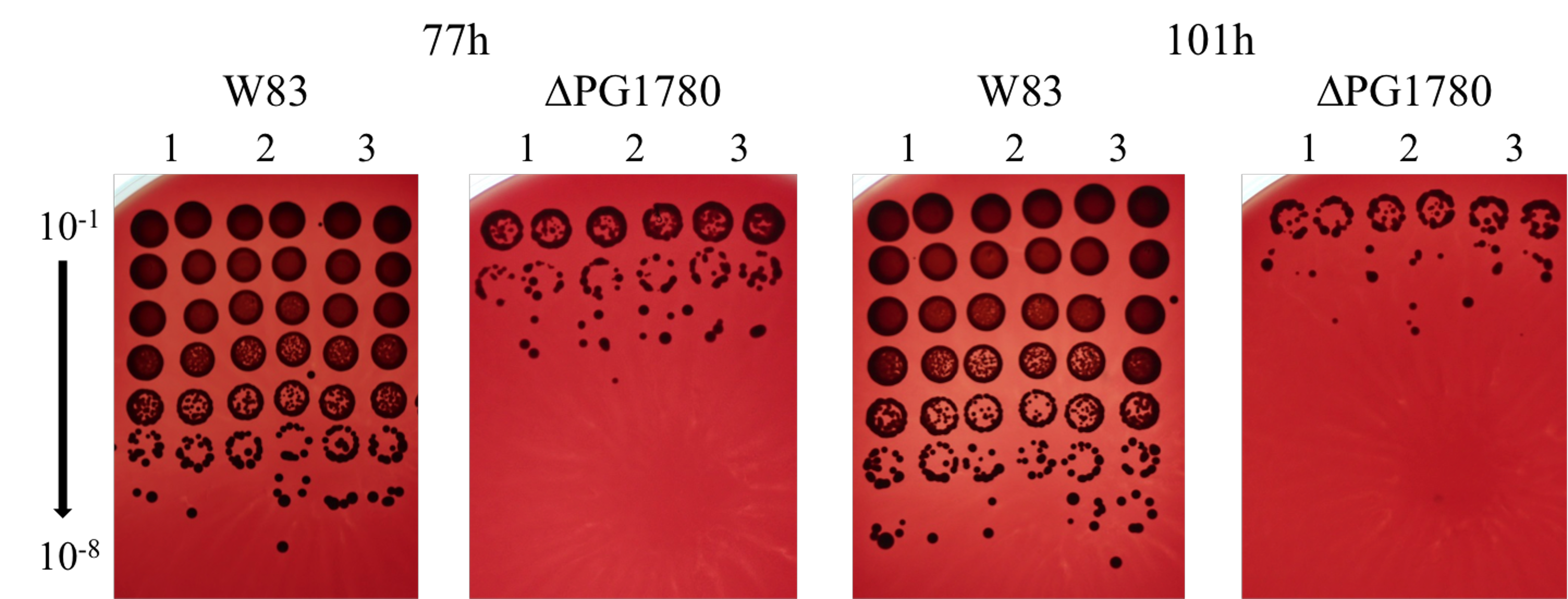

Supplement: Supplementary file 2 [file Data_Sheet_1.DOCX]
